# Proangiogenic effects of soluble α-Klotho on systemic sclerosis dermal microvascular endothelial cells

**DOI:** 10.1186/s13075-017-1233-0

**Published:** 2017-02-10

**Authors:** Celestina Mazzotta, Mirko Manetti, Irene Rosa, Eloisa Romano, Jelena Blagojevic, Silvia Bellando-Randone, Cosimo Bruni, Gemma Lepri, Serena Guiducci, Lidia Ibba-Manneschi, Marco Matucci-Cerinic

**Affiliations:** 1 Department of Experimental and Clinical Medicine, Division of Rheumatology, University of Florence, AOUC, Largo Brambilla 3, 50134 Florence, Italy; 2 Department of Experimental and Clinical Medicine, Section of Anatomy and Histology, University of Florence, Largo Brambilla 3, 50134 Florence, Italy

**Keywords:** α-Klotho, Endothelial cells, Angiogenesis, Systemic sclerosis

## Abstract

**Background:**

Systemic sclerosis (SSc) is characterized by endothelial cell (EC) apoptosis, impaired angiogenesis and peripheral microvasculopathy. Soluble α-Klotho (sKl) is a pleiotropic molecule with multiple effects on ECs, including antioxidant and vasculoprotective activities. On the EC surface, sKl interacts with vascular endothelial growth factor (VEGF) receptor-2 (VEGFR-2) and transient receptor potential canonical-1 (TRPC-1) cation channel to control EC homeostasis. Here, we investigated whether sKl might act as a protective factor to improve angiogenesis in dermal microvascular endothelial cells (MVECs) from SSc patients (SSc-MVECs).

**Methods:**

Wound healing assay was performed on healthy dermal MVECs (H-MVECs) challenged with sera from healthy controls or SSc patients with or without the addition of sKl. Capillary morphogenesis on Matrigel was assessed in H-MVECs and SSc-MVECs at basal conditions and treated with sKl, as well as in H-MVECs challenged with healthy or SSc sera in presence or absence of sKl. The expression of α-Klotho, VEGF_165_b, VEGFR-2, TRPC-1, Ki67 and active caspase-3 in H-MVECs and SSc-MVECs was investigated by western blotting. Immunostaining for α-Klotho was performed in H-MVECs and SSc-MVECs, and in healthy and SSc skin sections.

**Results:**

Treatment with sKl effectively counteracted the inihibitory effects of SSc sera on wound healing ability and angiogenic performance of H-MVECs. The addition of sKl significantly improved angiogenesis and maintained over time capillary-like tube formation in vitro by SSc-MVECs. Stimulation of SSc-MVECs with sKl resulted in the upregulation of the proliferation marker Ki67 in parallel with the downregulation of proapoptotic active caspase-3. The expression of α-Klotho was significantly lower in SSc-MVECs than in H-MVECs. The expression of TRPC-1 was also significantly decreased, while that of VEGFR-2 and VEGF_165_b was significantly increased, in SSc-MVECs compared with H-MVECs. Challenge with sKl either significantly increased TRPC-1 or decreased VEGF_165_b in SSc-MVECs. Ex vivo analyses revealed that α-Klotho immunostaining was almost absent in the dermal microvascular network of SSc skin compared with control skin.

**Conclusions:**

Our findings provide the first evidence that α-Klotho is significantly decreased in the microvasculature in SSc skin and that sKl administration may effectively improve SSc-MVEC functions in vitro by acting as a powerful proangiogenic factor.

## Background

Systemic sclerosis (SSc) is an autoimmune connective tissue disease characterized by alterations in the microcirculation, deregulation of immune response and fibrosis of the skin and internal organs [1]. According to the degree of skin involvement, SSc is categorized into limited and diffuse cutaneous SSc subsets (lcSSc and dcSSc, respectively) [2, 3]. Vascular damage occurs early in the course of SSc, with chronic tissue ischemia and lack of compensatory angiogenesis, progressing until the loss of dermal capillaries and formation of non-healing digital ulcers [4].

Angiogenesis, a process controlled by a tight balance between proangiogenic and anti-angiogenic signals, is essential for wound healing [5]. Among proangiogenic molecules, vascular endothelial growth factor (VEGF) has a pivotal role in postnatal neovascularization, which comprises angiogenesis and vasculogenesis [6–8]. Indeed, angiogenesis is the formation of new blood vessels by sprouting of pre-existing mature endothelial cells (ECs), while vasculogenesis is a process involving the recruitment and differentiation of bone marrow (BM)-derived endothelial precursor cells (EPCs) [9, 10]. In this context, several studies have reported that VEGF levels are significantly elevated in serum in SSc despite the lack of adaptive angiogenesis [11–15]. Moreover, microvascular ECs (MVECs) isolated from skin in SSc display a defective angiogenic capacity in vitro, including impaired proliferation, migration and capillary-like tube formation, even in response to exogenous VEGF stimulation [4].

**Fig. 1:**
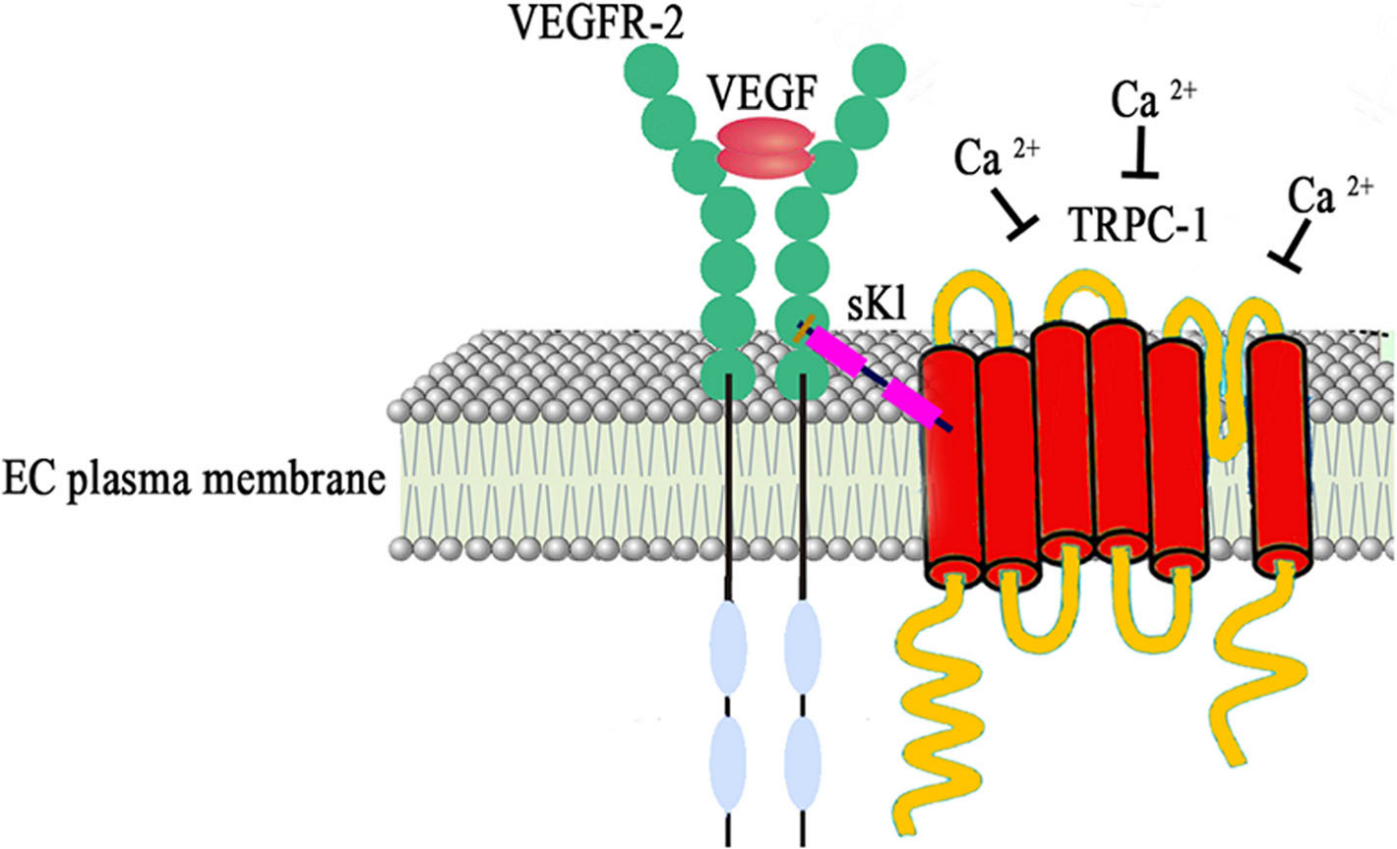
Schematic representation of the molecular complex formed by vascular endothelial growth factor (*VEGF*)-bound VEGF receptor-2 (*VEGFR-2*), transient receptor potential canonical-1 (*TRPC-1*) and soluble α-Klotho (*sKl*) on the surface of endothelial cells (*ECs*). This molecular complex regulates the influx of Ca^2+^ and the permeability of the plasma membrane contributing to the maintenance of EC integrity

A recent study demonstrated that soluble α-Klotho (sKl) protein interacts directly both with VEGF receptor-2 (VEGFR-2) and transient receptor potential canonical-1 (TRPC-1) cation channel on the surface of ECs [16]. The resulting heterotrimeric molecular complex is incorporated into ECs in response to VEGF stimulation and is involved in stabilizing the entry of Ca^2+^ in order to maintain EC integrity [16] (Fig. 1).

The α*-Klotho* gene encodes a type I single-pass-transmembrane protein composed of a large extracellular domain (130 kDa), a transmembrane domain and a very short intracellular domain (10 amino acids) [17–19]. The extracellular domain has homologies with the family of 1-glycosidases and is subjected to ectodomain shedding. As a result, the entire extracellular domain is released into the extracellular space and is detectable in blood, urine and cerebrospinal fluid [20–22]. Therefore, α-Klotho protein has transmembrane (tKl) and soluble circulating (sKl) forms with different properties.

The full-length tKl functions as an obligatory co-receptor for fibroblast growth factor 23 (FGF23) forming a complex with FGF receptors and FGF23 that regulates phosphate homeostasis by inhibiting 1,25(OH)_2_ vitamin D_3_ synthesis and inducing phosphaturia [23–25]. sKl functions instead as a humoral factor with pleiotropic activities, such as the suppression of growth factor signaling and oxidative stress and the regulation of ion channels and transporters [26, 27]. Moreover, sKl increases endothelial nitric oxide (NO) production, improves endothelium-dependent vasodilatation, induces resistance to lipid peroxidation and exerts anti-inflammatory and anti-cancer effects [28]. In addition, sKl negatively regulates insulin/insulin-like growth factor-1 signaling with consequent inhibition of cellular senescence through the inactivation of Akt and the induction of p53 and p21 expression [20].

Insulin, by stimulating the proteolytic activities of a disintegrin and metalloprotease (ADAM)-10 and ADAM-17, may enhance tKl shedding and the release of the extracellular domain of α-Klotho [29]. Circulating α-Klotho may derive also by alternative mRNA splicing of exon 3 with generation of a different transcript called secreted α-Klotho, which has a half-length of the full-length transcript [17, 18, 30] and is believed to act as a hormone regulating the functions of cells or tissues that do not express α-Klotho. On ECs sKl plays the role of antioxidant with the ability to downregulate generation of reactive oxygen species (ROS) and mediate vasculoprotective effects [31, 32].

Based on these facts, the aim of the present study was to examine whether sKl could exert positive effects on dermal MVECs from patients with SSc by promoting in vitro angiogenesis.

## Methods

### Patients and controls

Serum samples were obtained from five patients with lcSSc (n = 3) or dcSSc (n = 2) (median age 61 years, range 35–76 years) [2] and from five age-matched and sex-matched healthy individuals. The patients enrolled were not on corticosteroids, immunosuppressants or other disease-modifying drugs. Peripheral blood samples were collected without any additive, left to clot for 30 minutes before centrifugation at 1500 g for 15 minutes, and serum was collected and stored in aliquots at −80 °C until used.

Paraffin-embedded sections of lesional forearm skin biopsies were obtained from 15 patients with SSc (13 women and 2 men; n = 10 with lcSSc and n = 5 with dcSSc, median age 45.3 years, range 29–67 years, and median disease duration 5 years, range 1–14 years) and 13 age-matched and sex-matched healthy donors. The study was approved by the Ethical Committee of the Azienda Ospedaliero-Universitaria Careggi (AOUC), Florence, Italy, and all subjects provided written informed consent. The study was conducted in accordance with the Declaration of Helsinki.

### Isolation of dermal MVECs and cell culture

Dermal MVECs were isolated from skin biopsies from five patients with dcSSc (SSc-MVECs) and from five healthy subjects (H-MVECs). Briefly, skin samples were mechanically cleaned to remove the adipose and epidermal layers, in order to obtain a pure specimen of vascularized dermis, and were treated as described elsewhere [33]. The samples were placed at 37 °C in a humidified atmosphere with 5% CO_2_. After one day of culture in EC basal medium (EBM-2, catalog number LOCC3156; Euroclone, Milan, Italy) supplemented with 20% fetal bovine serum (FBS), 5 ng/ml H-epidermal growth factor (hEGF; Clonetics Corporation, San Diego, CA, USA), 1 μg/ml hydrocortisone acetate, 100 U/ml penicillin, 100 μg/ml streptomycin and 25 μg/ml amphotericin B without addition of further angiogenic growth factors, small colonies of polygonal elements were detected. Non-adherent cells were removed and fresh EC complete medium was added. To maintain optimal culture conditions, the medium was changed every third day, and after 2 weeks of primary culture a monolayer of cells was obtained. MVECs from primary cultures were further identified using immunomagnetic beads recognizing CD31. Isolated cells were purified as MVECs by labeling with anti-factor VIII-related antigen and anti-CD105, followed by reprobing with anti-CD31 antibodies. Dermal MVECs were maintained in EC complete medium and were used between the third and seventh passages in culture.

### In vitro wound healing assay

Wound healing assay was performed on confluent H-MVECs in basal condition and challenged with serum from healthy controls (n = 5) or patients with SSc (n = 5 (n = 3 with lcSSc and n = 2 with dcSSc)) with or without the addition of recombinant human sKl protein (5 ng/ml) (Active Human α-Klotho protein fragment, catalog number ab84072; Abcam, Cambridge, UK). H-MVECs were seeded onto 6-well tissue culture plates and cultured in EBM-2 medium with 10% FBS until 80–90% of confluence. The cells were then rinsed with phosphate-buffered saline (PBS) and starved in low serum medium (0.5% FBS) overnight. The day after, a wound was made on the cellular monolayer using a sterile 200-μl pipet tip followed by a wash with EBM-2 basal medium to remove detached cells. Wound healing capacity was assessed by capturing phase-contrast images of the wounded area at the beginning (T0) and at 24 hours (T24) under a Leica inverted microscope (Leica Microsystems, Mannheim, Germany), and comparing the images to quantify the migration rate of the cells after wounding. All experimental conditions were performed in triplicate.

### In vitro capillary morphogenesis assay

In vitro capillary morphogenesis assay was performed in 96-well plates covered with Matrigel (BD Biosciences, San Jose, CA, USA). Matrigel (50 μl; 10–12 mg/ml) was pipetted into culture wells and polymerized for 30 minutes at 37 °C, as described elsewhere [13]. H-MVECs and SSc-MVECs (30 × 10^3^ cells/well) were incubated in basal EBM-2 medium containing 10% FBS or serum from five patients with SSc or five healthy subjects, with or without the addition of recombinant human sKl protein (5 ng/ml). Stimulation with recombinant human VEGF_165_ (10 ng/ml) (R&D Systems, Minneapolis, MN, USA) was used as positive control of angiogenesis. All conditions were performed in triplicate and the plates were photographed at 24 and 48 hours. Results were quantified at the same time by measuring the percent field occupancy of capillary projections, as determined by image analysis. Six to nine photographic fields from three plates were scanned for each point.

### Western blotting

H-MVECs and SSc-MVECs were cultured until confluence, detached with trypsin-EDTA, washed with PBS, pelleted and subjected to total protein extraction. In some experimental conditions, H-MVECs and SSc-MVECs were stimulated with recombinant human sKl protein (5 ng/ml) (catalog number ab84072; Abcam) for 24 and 48 hours before protein extraction. Twenty-five micrograms of total proteins were electrophoresed on NuPAGE 4 to 12% Bis-Tris Gel (Invitrogen, Carlsbad, CA, USA) and blotted onto polyvinylidene difluoride membranes (Invitrogen). The membranes were blocked with blocking solution included in the Western Breeze Chromogenic Western Blot Immunodetection Kit (Invitrogen) for 40 minutes at room temperature on a rotary shaker and subsequently incubated for 1 hour at room temperature with rabbit monoclonal anti-human α-Klotho (1:500 dilution; catalog number ab181373; Abcam), mouse monoclonal anti-human VEGF_165_b (1:500 dilution; catalog number ab14994; Abcam), rabbit polyclonal anti-human VEGFR-2 (1:100 dilution; catalog number ab45010; Abcam), rabbit monoclonal anti-human TRPC-1 (1:1000 dilution; catalog number ab51255; Abcam), rabbit monoclonal anti-human Ki67 (1:1000 dilution; catalog number ab92742; Abcam), rabbit polyclonal anti-human caspase-3 (1:1000 dilution; catalog number 9662; Cell Signaling Technology, Danvers, MA, USA), and rabbit polyclonal anti-human α-tubulin (1:1000 dilution; catalog number ab18251; Abcam) antibodies, assuming α-tubulin as control invariant protein. Immunodetection was performed as described in the Western Breeze Chromogenic Immunodetection protocol (Invitrogen). ImageJ software was used for densitometric analysis of the bands and the values were normalized to α-tubulin.

### Immunohistochemical analysis

After deparaffinization and rehydration, skin sections (5 μm thick) were boiled for 10 minutes in sodium citrate buffer (10 mM, pH 6.0) for antigen retrieval and treated with 3% H_2_O_2_ in methanol for 15 minutes at room temperature to block endogenous peroxidase activity. Sections were then washed and incubated with Ultra V block (UltraVision Large Volume Detection System Anti-Polyvalent, HRP, catalog number TP-125-HL; LabVision, Fremont, CA, USA) for 10 minutes at room temperature according to the manufacturer’s protocol. After blocking non-specific site binding, slides were incubated overnight at 4 °C with rabbit monoclonal anti-human α-Klotho antibody (1:20 dilution; catalog number ab181373; Abcam) diluted in 1% bovine serum albumin (BSA) in PBS.

The day after, tissue sections were washed three times in PBS and incubated with biotinylated secondary antibodies (UltraVision Large Volume Detection System Anti-Polyvalent, HRP; LabVision) for 10 minutes at room temperature. Subsequently, the slides were washed three times in PBS and incubated with streptavidin peroxidase (UltraVision Large Volume Detection System Anti-Polyvalent, HRP; LabVision) for 10 minutes at room temperature. Immunoreactivity was developed using 3-amino-9-ethylcarbazole (AEC kit, catalog number TA-125-SA; LabVision) as chromogen. Skin sections were finally counterstained with Mayer’s hematoxylin (Bio-Optica, Milan, Italy), washed, mounted in an aqueous mounting medium and observed under a Leica DM4000 B microscope (Leica Microsystems). Sections not exposed to primary antibodies or incubated with isotype-matched and concentration-matched non-immune rabbit IgG (Sigma-Aldrich, St. Louis, MO, USA) were included as negative controls for antibody specificity.

Light microscopy images were captured with a Leica DFC310 FX 1.4-megapixel digital color camera equipped with the Leica software application suite LAS V3.8 (Leica Microsystems). α-Klotho immunostaining was quantified in a semi-quantitative manner, where 0 indicates no staining, 1 indicates weak staining, 2 indicates moderate staining and 3 indicates strong staining in microvessels at eight randomly chosen high-power fields (×40 original magnification) per sample. Two different examiners performed blinded evaluation. When there was interobserver disagreement, the specimen was reviewed again by both observers and the disagreement resolved.

### Immunocytochemical analysis

MVECs were seeded onto glass coverslips, grown to 70% confluence, fixed with 3.7% buffered paraformaldehyde and permeabilized with 0.1% Triton X-100 in PBS. Slides were then washed, treated with 3% H_2_O_2_ in PBS for 15 minutes at room temperature and subsequently blocked with Ultra V block (UltraVision Large Volume Detection System Anti-Polyvalent, HRP; LabVision) for 10 minutes. Cells were incubated overnight at 4 °C with rabbit monoclonal anti-human α-Klotho antibody (catalog number ab181373; Abcam) at 1:20 dilution in 1% BSA in PBS, followed by incubation with biotinylated secondary antibodies and streptavidin peroxidase (UltraVision Large Volume Detection System Anti-Polyvalent, HRP; LabVision) at room temperature.

Immunoreactivity was developed with 3-amino-9-ethylcarbazole (AEC kit; LabVision). Irrelevant isotype-matched and concentration-matched rabbit IgG (Sigma-Aldrich) were used as negative controls. Nuclei were counterstained with Mayer’s hematoxylin (Bio-Optica). Immunolabeled cells were examined with a Leica DM4000 B microscope (Leica Microsystems) and photomicrographs were captured with a Leica DFC310 FX 1.4-megapixel digital colour camera equipped with the Leica software application suite LAS V3.8 (Leica Microsystems).

### Statistical analysis

Data are expressed as the mean ± standard deviation (SD) or the mean ± standard error of the mean (SEM). Student’s *t* test and Tukey’s multiple comparisons test were used where appropriate for statistical evaluation of the differences between independent groups. A *p* value less than 0.05 was considered statistically significant.

## Results

### Effects of sKl on wound healing capacity of MVECs

**Fig. 2:**
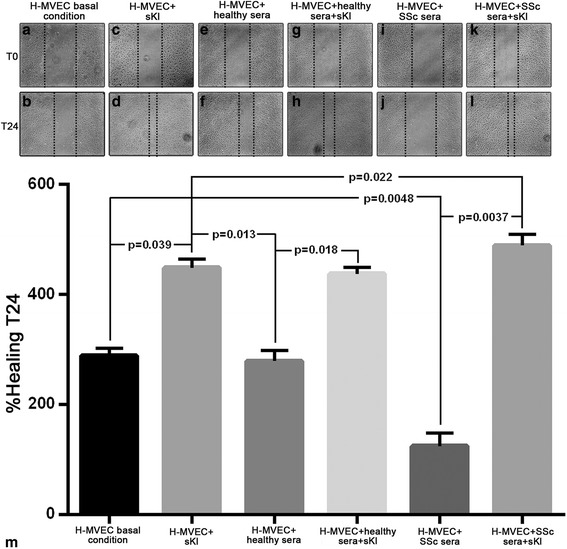
Soluble α-Klotho (*sKl*) improves the wound healing capacity of healthy dermal microvascular endothelial cells (*H-MVECs*) and counteracts the inhibitory effects of serum from patients with systemic sclerosis (*SSc*). Wound healing capacity of H-MVECs was assayed at basal conditions and in the presence of serum from healthy controls (n = 5) and patients with SSc (n = 5), with or without the addition of sKl. **a**–**l** Representative images of the wounded area at 0 hours and 24 hours after scratching. **m** Quantitative analysis of the percentage of wound repair. Data are means ± SD of three independent experiments performed in triplicate with each one of the five H-MVEC lines. Tukey’s multiple comparisons test was used for statistical analysis; *p* values are indicated

The in vitro wound healing assay was performed to evaluate the ability of H-MVECs to repair the injured area in standard conditions or under challenge with serum from healthy subjects or patients with SSc, in the presence or absence of sKl (Fig. 2a–l). After 24 hours, H-MVECs cultured in standard conditions and challenged with sKl had increased ability to repair the wounded area when compared to basal H-MVECs without sKl (*p* = 0.039) (Fig. 2b, d, m). Moreover, the addition of sKl to H-MVECs treated with healthy serum improved the wound healing process compared with H-MVECs treated with healthy serum alone (*p* = 0.018) (Fig. 2f, h, m). H-MVECs challenged with serum from patients with SSc lost the ability to repair the wounded area (Fig. 2j) in respect to H-MVECs at basal condition and H-MVECs cultured with healthy serum (*p* = 0.0048 and *p* = 0.0008, respectively) (Fig. 2b, f, m). Interestingly, the administration of sKl to H-MVECs challenged with serum from patients with SSc significantly improved wound healing compared to H-MVECs treated with serum from patients with SSc alone (*p* = 0.0037) (Fig. 2j, l, m) and compared to H-MVECs with sKl or H-MVECs treated with healthy serum in the presence of sKl (*p* = 0.022 and *p* = 0.031, respectively) (Fig. 2d, h, m).

These results suggest that sKl acts as a powerful pro-reparative factor exhibiting the most significant effects in a pathological microenvironment such as in the presence of serum from patients with SSc, by improving cell migration and cell growth toward the center of the wound. We also observed that H-MVECs cultured in standard condition or with healthy serum in the presence of sKl had a high proliferation rate in the first 24 hours, and subsequently the cells detached from the culture plate, and after 48 hours only 30% of the cells were still adherent to the plate (data not shown), suggesting that sKl may contribute to regulate cell overgrowth over time in normal conditions, while in pathological conditions it may foster and sustain reparative processes.

### Effects of sKl on capillary-like tube formation

**Fig. 3:**
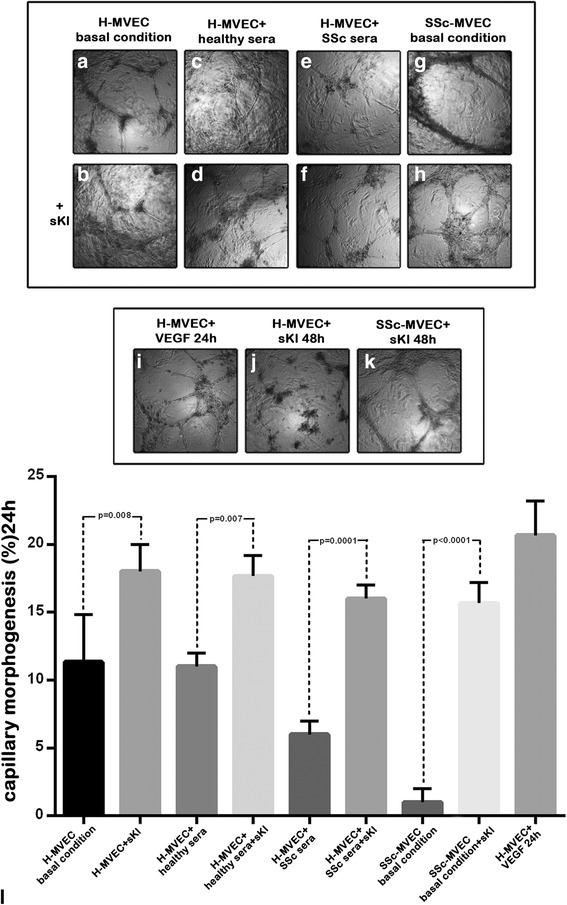
Effects of soluble α-Klotho (*sKl*) on the ability of dermal microvascular endothelial cells from healthy controls (*H-MVECs*) and patients with systemic sclerosis (SSc) (*SSc-MVECs*) to form capillary-like tubes on Matrigel. In vitro capillary morphogenesis of H-MVECs was evaluated at basal conditions and after challenge with serum from healthy controls (n = 5) and patients with SSc (n = 5), with or without the addition of sKl. Stimulation with vascular endothelial growth factor (*VEGF*)_165_ was included as a positive control for angiogenesis. In vitro capillary morphogenesis of SSc-MVECs was evaluated at basal conditions and after stimulation with sKl. **a**–**i** Representative images of the capillary network formed on Matrigel at 24 hours after plating are shown for each experimental point. **j**, **k** Representative images of H-MVECs (**j**) and SSc-MVECs (**k**) stimulated with sKl at 48 hours from plating on Matrigel. **l** Quantitative analysis of capillary morphogenesis on Matrigel as percent field occupancy of capillary projections at 24 hours after plating. Data are means ± SD of three independent experiments performed in triplicate with each one of the five H-MVEC and five SSc-MVEC lines. Tukey’s multiple comparisons test was used for statistical analysis; *p* values are indicated

As previously demonstrated [34, 35], at basal conditions the ability of H-MVECs to form capillary-like tubular structures after 24 hours of seeding on Matrigel was significantly greater compared with SSc-MVECs (*p* < 0.0001) (Fig. 3a, g). Furthermore, in the first 24 hours, the addition of sKl significantly increased the angiogenic ability of H-MVECs compared to basal conditions (*p* = 0.008) (Fig. 3a, b, l), but after 48 hours the tubular structures were degenerated (Fig. 3j). For SSc-MVECs, the addition of sKl significantly improved angiogenesis compared with cells at basal conditions (*p* < 0.0001), by over time restoring and maintaining the sprouting of capillary-like tubes organized into a honeycomb morphological pattern (Fig. 3g, h, k, l). Moreover, the angiogenic response of H-MVECs cultured with serum from patients with SSc was impaired in respect to either H-MVECs at basal conditions or H-MVECs challenged with healthy serum (*p* = 0.04 for both comparisons) (Fig. 3a, c, e). The negative effect of serum from patients with SSc on H-MVECs was reversed by the addition of sKl as demonstrated by the production of an endothelial network comparable to those observed in basal H-MVECs and H-MVECs cultured with healthy serum (Fig. 3f).

Consistent with the wound healing assay data, sKl had a different effect in normal or pathological conditions. In fact, in H-MVECs the addition of sKl induced angiogenesis up to 24 hours, while at 48 hours it had an inhibitory effect on cell proliferation by regulating cell overgrowth. Conversely, in SSc-MVECs sKl ameliorated the production of elongated processes and improved the organization of cellular branching cords by maintaining its positive effect over time.

### Differential regulation of proliferative and apoptotic pathways by administration of sKl to H-MVECs and SSc-MVECs

**Fig. 4:**
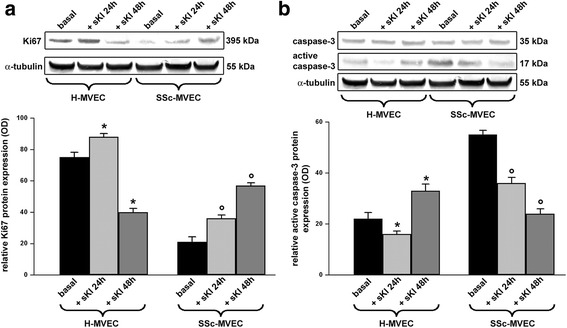
Expression of the proliferative marker Ki67 and proapoptotic active caspase-3 in dermal microvascular endothelial cells from healthy controls (*H-MVECs*) and patients with systemic sclerosis (SSc) (*SSc-MVECs*). **a**, **b** Western blotting of total protein extracts from H-MVECs and SSc-MVECs at basal condition and treated with recombinant human soluble α-Klotho (*sKl*) protein for 24 and 48 hours assayed with anti-Ki67 (**a**) and anti-caspase 3 (**b**) antibodies. Representative immunoblots are shown. The active fragment of casapase-3 is identified at the expected molecular weight of 17 kDa. The densitometric analysis of the bands normalized to α-tubulin is reported in the histograms. Data are mean ± SD of optical density (*OD*) in arbitrary units. Student’s *t* test was used for statistical analysis; *p < 0.05 versus basal H-MVECs, °*p* < 0.05 versus basal SSc-MVECs. Results are representative of three independent experiments performed with each one of the five H-MVEC and five SSc-MVEC lines

In order to investigate the effects of sKl administration on H-MVEC and SSc-MVEC proliferation and apoptosis, we analyzed the protein expression levels of the proliferative marker Ki67 and proapoptotic active caspase-3 at basal condition and after stimulation with sKl for 24 and 48 hours. As displayed in Fig. 4a, compared with the basal condition, challenge with sKl for 24 hours increased Ki67 expression levels in H-MVECs (*p* < 0.05). Conversely, Ki67 protein levels were significantly decreased in H-MVECs treated with sKl for 48 hours in respect to basal H-MVECs (*p* < 0.05) (Fig. 4a).

Ki67 protein expression was strongly reduced in basal SSc-MVECs compared with basal H-MVECs (Fig. 4a). The addition of sKl to SSc-MVECs resulted in significant progressive upregulation of Ki67 up to 48 hours (*p* < 0.05 for either 24-hour or 48-hour treatment versus the basal condition) (Fig. 4a).  As shown in Fig. 4b, 24-hour stimulation of H-MVECs with sKl significantly reduced active caspase-3 (*p* < 0.05 versus basal H-MVECs), while challenge with sKl for 48 hours resulted in significant upregulation of active caspase-3 (*p* < 0.05 versus basal H-MVECs). At baseline, active caspase-3 protein levels were significantly higher in SSc-MVECs compared with H-MVECs (Fig. 4b). Treatment of SSc-MVECs with sKl led to a significant progressive decrease in active caspase-3 up to 48 hours (*p* < 0.05 for either 24-hour or 48-hour treatment versus the basal condition) (Fig. 4b).

### Expression of α-Klotho in skin biopsies

**Fig. 5:**
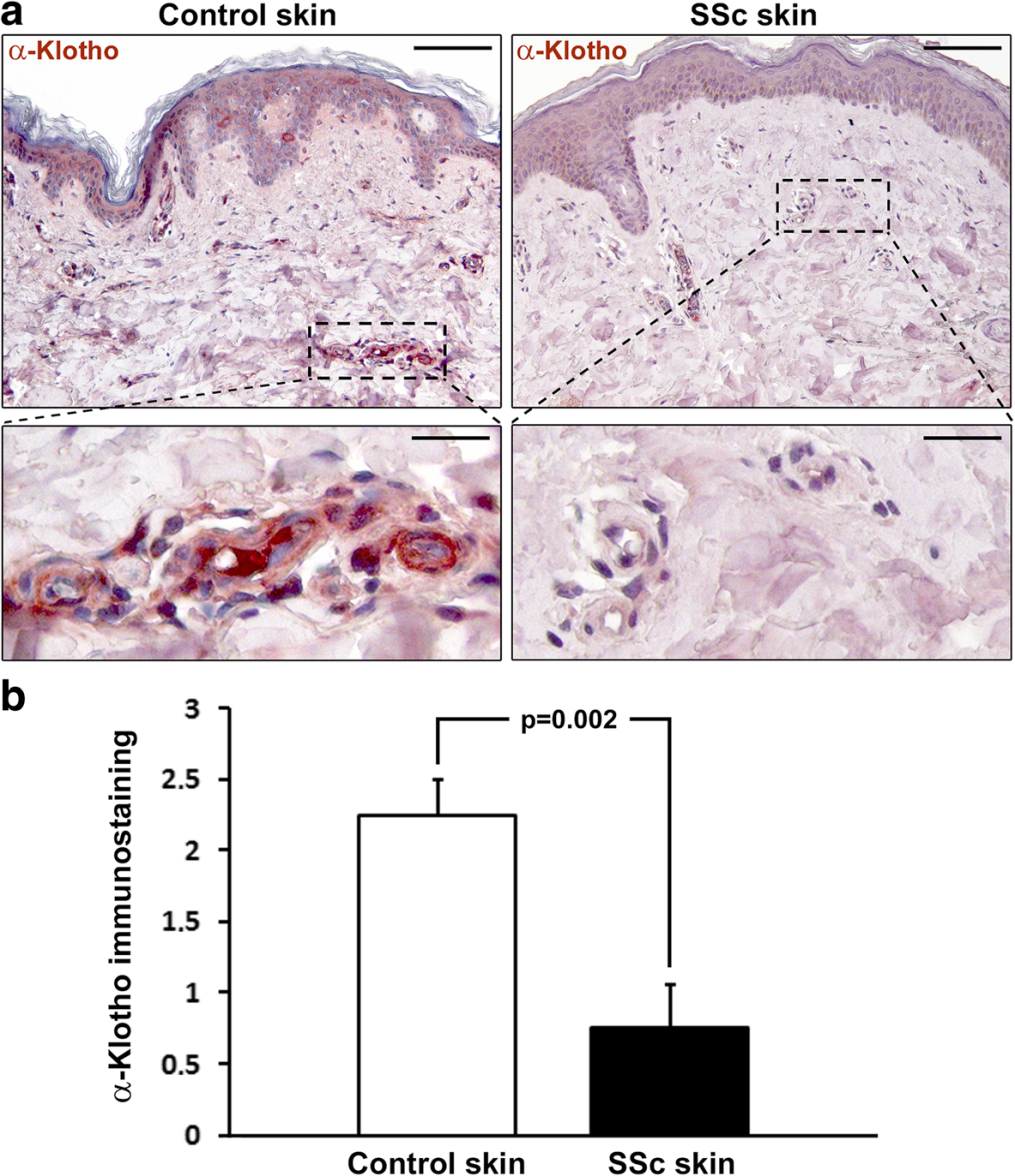
Decreased expression of α-Klotho in the skin of patients with systemic sclerosis (*SSc*). **a** Representative microphotographs of skin sections from healthy controls and patients with SSc immunostained with rabbit monoclonal anti-human α-Klotho antibody and counterstained with hematoxylin. *Boxed area* (*upper panels*) is shown at higher magnification in the respective *lower panels*. α-Klotho immunostaining is decreased in dermal microvessels of the skin in SSc compared with healthy control skin. *Scale bar* 100 μm (*upper panels*) and 20 μm (*lower panels*). **b** Semi-quantitative analysis of α-Klotho immunostaining in dermal microvessels. Data are mean ± SEM of immunostaining score performed on skin sections from eight patients with SSc and eight healthy controls. Student’s *t* test was used for statistical analysis; the *p* value is indicated

The expression of α-Klotho in skin biopsies from patients with SSc and healthy controls was evaluated by immunohistochemical staining. α-Klotho immunostaining was strongly decreased in different cellular components of the skin in SSc, including dermal fibroblasts, as compared with healthy skin (Fig. 5a). Moreover, α-Klotho immunostaining was almost absent in the dermal microvascular network of the skin in SSc compared with healthy controls (Fig. 5a). As displayed in Fig. 5b, semi-quantitative analysis of immunostaining revealed significantly lower expression of α-Klotho in the dermal microvessels in patients with SSc compared with controls (*p* = 0.002).

### Protein expression levels of α-Klotho, VEGF_165_b, VEGFR-2 and TRPC-1 in MVECs

**Fig. 6:**
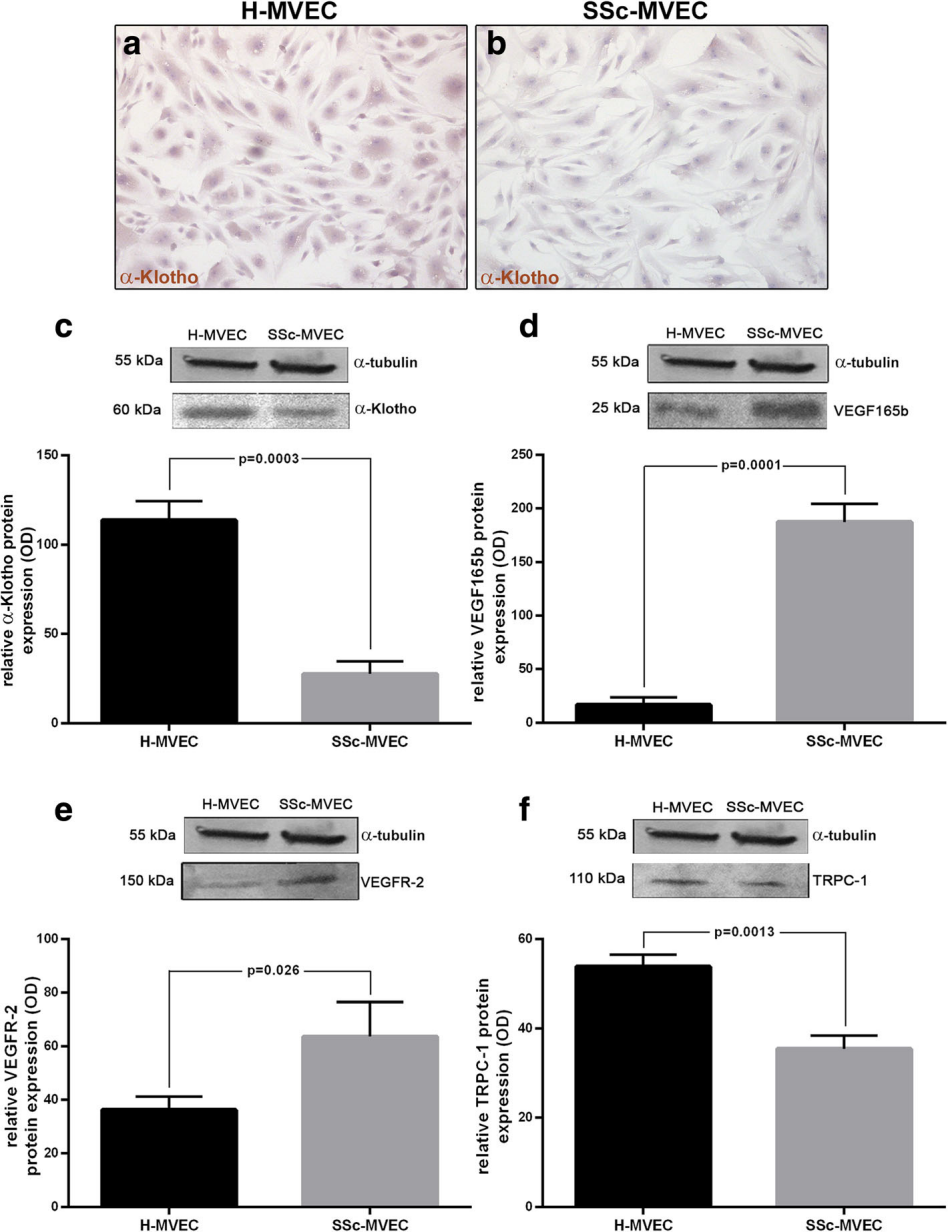
Expression of α-Klotho, vascular endothelial growth factor (*VEGF*)_165_b, VEGF receptor-2 (*VEGFR-2*) and transient receptor potential canonical-1 (*TRPC-1*) in dermal microvascular endothelial cells from healthy controls (*H-MVECs*) and patients with systemic sclerosis (SSc) (*SSc-MVECs*). **a**, **b** Representative microphotographs of immunocytochemical staining for α-Klotho in H-MVECs (**a**) and SSc-MVECs (**b**) detected with biotinylated secondary antibodies and streptavidin peroxidase. **c**-**f** Protein lysates from H-MVECs and SSc-MVECs were subjected to western blotting analysis using anti-α-Klotho (**c**), anti-VEGF_165_b (**d**), anti-VEGFR-2 (**e**) and anti-TRPC-1 (**f**) antibodies. Representative immunoblots are shown. The densitometric analysis of the bands normalized to α-tubulin is reported in the histograms. Data are mean ± SD of optical density (*OD*) in arbitrary units. Student’s *t* test was used for statistical analysis; *p* values are indicated in each panel. Results are representative of three independent experiments performed with each one of the five H-MVEC and five SSc-MVEC lines

It has been demonstrated that sKl plays a pivotal role in the maintenance of EC integrity through direct interaction with VEGFR-2 and TRPC-1 cation channel and consequent formation of a heterotrimeric complex, which is incorporated into ECs in response to VEGF [16]. As the expression levels of the anti-angiogenic VEGF_165_b isoform have been reported to be selectively increased in SSc-MVECs compared with H-MVECs [13], and considering that there are no anti-human α-Klotho antibodies suitable for a co-immunoprecipitation assay, we evaluated the expression of α-Klotho, VEGF_165_b, VEGFR-2 and TRPC-1 proteins in H-MVECs and SSc-MVECs (Fig. 6a–f). In protein lysates from SSc-MVECs, the expression of α-Klotho was significantly decreased compared to H-MVECs (*p* = 0.0003) (Fig. 6c). Immunocytochemical analysis confirmed these results showing lower expression of α-Klotho at cytoplasmic subcellular level in SSc-MVECs compared with H-MVECs (Fig. 6a and b). Moreover, TRPC-1 protein expression was significantly lower in SSc-MVECs than in H-MVECs (*p* = 0.0013), while protein levels of VEGFR-2 and VEGF_165_b were both significantly higher in SSc-MVECs compared to H-MVECs (*p* = 0.026 and *p* = 0.0001, respectively) (Fig. 6d, e, f).

**Fig. 7:**
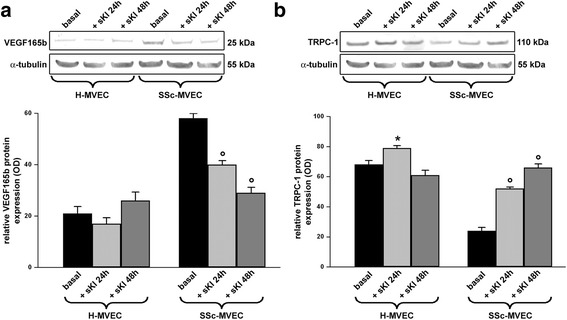
Expression of vascular endothelial growth factor (*VEGF*)_165_b and transient receptor potential canonical-1 (*TRPC-1*) in dermal microvascular endothelial cells from healthy controls (*H-MVECs*) and systemic sclerosis (SSc) patients (*SSc-MVECs*). **a**, **b** Western blotting of total protein extracts from H-MVECs and SSc-MVECs at basal condition and treated with recombinant human soluble α-Klotho (*sKl*) protein for 24 and 48 hours assayed with anti-VEGF_165_b (**a**) and anti-TRPC-1 (**b**) antibodies. Representative immunoblots are shown. The densitometric analysis of the bands normalized to α-tubulin is reported in the histograms. Data are mean ± SD of optical density (*OD*) in arbitrary units. Student’s *t* test was used for statistical analysis; *p < 0.05 versus basal H-MVECs, °*p* < 0.05 versus basal SSc-MVECs. Results are representative of three independent experiments performed with each one of the five H-MVEC and five SSc-MVEC lines

As we have shown that stimulation with sKl may ameliorate in vitro angiogenesis in SSc-MVECs, we further investigated whether sKl could mediate these proangiogenic effects though the modulation of VEGF_165_b and TRPC-1 expression. As displayed in Fig. 7a, challenge of SSc-MVECs with sKl resulted in a significant progressive decrease in VEGF_165_b protein up to 48 hours (*p* < 0.05 for either 24-hour or 48-hour treatment versus the basal condition). Moreover, there was a significant progressive increase in TRPC-1 in SSc-MVECs challenged with sKl (*p* < 0.05 for either 24-hour or 48-hour treatment versus basal condition) (Fig. 7b). As far as H-MVECs are concerned, stimulation with sKl did not result in any significant changes in VEGF_165_b protein expression either at 24 or 48 hours, while TRPC-1 was significantly upregulated only after 24-hour treatment (*p* < 0.05 versus basal condition) (Fig. 7a, b).

These results suggest that either the downregulation of α-Klotho and TRPC-1 or the preferential binding of the upregulated anti-angiogenic VEGF_165_b isoform to VEGFR-2 may prevent the formation of the VEGF_165_/VEGFR-2/TRPC-1/sKl molecular complex on the plasma membrane of SSc-MVECs, with consequent negative effects on cellular homeostasis and angiogenesis. According to our western blotting data, the proangiogenic effects exerted over time on SSc-MVECs by the addition of sKl might in part be explained by sKl-induced downregulation of VEGF_165_b and parallel upregulation of TRPC-1.

## Discussion

This is the first report to investigate the possible protective and proangiogenic effects of sKl on vascular ECs from patients with SSc. Indeed, we have shown that sKl improves SSc-MVEC angiogenesis in vitro, with a robust positive effect persisting over time. In particular, by restoring vessel sprouting, treatment with sKl enhances the number and improves the morphology of capillary-like tubes formed by SSc-MVECs in vitro. On the contrary, we observed that in a physiological condition mirrored in vitro by H-MVECs, sKl stimulates angiogenesis only transiently, while inducing cell detachment with consequent vessel regression and dissociation over time.

This dualistic behavior of α-Klotho might depend on its ability to influence the activation of the normal cell cycle regulatory pathways, including p53/p21. Indeed, several reports have demonstrated that sKl attenuates cellular apoptosis and senescence in vascular cells via mitogen-activated kinase and extracellular signal-regulated kinase pathways [36, 37]. In fact, by regulating p53-mediated cell cycle arrest in the transition from G1 to S phase of cell replication [38], sKl may influence the p53/p21 pathway. In this context, our in vitro observations suggest that sKl may act preferentially as a protective and proangiogenic factor in pathological conditions, while exerting a regulatory role to maintain normal cell growth in physiological conditions. Indeed, a tight balance between survival and death signaling determines the cell fate [39], and continuous stimulation by growth factors to enhance proliferation is perceived by normal cells as a negative signal.

In all cell types the activation of apoptosis-regulatory genes is a physiological mechanism to control cell proliferation, and the deregulation of this complex mechanism is the primary cause of neoplastic transformation. Cultured non-transformed cells have a limited proliferative capacity, known as the Hayflick limit, which results in an irreversible proliferative arrest, called cellular senescence [40]. Indeed, according to this view, our Ki67 and active caspase-3 western blotting data show that while stimulation with sKl induces proliferation and inhibits apoptosis of SSc-MVECs over time, such pro-proliferative and anti-apoptotic effects are only transient in H-MVECs, being in fact rapidly followed by cell proliferation arrest and increased apoptosis.

Our ex vivo data demonstrated decreased expression of α-Klotho in skin biopsies from patients with SSc, in particular at microvascular level. In addition, in cultured SSc-MVECs α-Klotho was downregulated compared with H-MVECs, which instead exhibited strong expression of α-Klotho at cytoplasmic level. Interestingly, western blotting experiments on MVEC lysates confirmed the presence of a 60-kDa α-Klotho isoform that could derive from alternative splicing of exon 3; considering that, to the best of our knowledge, only the premature form of α-Klotho (130 kDa) was previously found in the endoplasmic reticulum and Golgi apparatus [22, 41].

In vascular ECs, α-Klotho upregulates the production of NO with protective effects against cell dysfunction [42, 43]. Of note, experimental models of parabiosis (i.e. the surgical connection of two animals to allow exchange of humoral factors) between α*-klotho* heterozygous and wild-type mice have shown complete recovery of endothelial function in α*-klotho* heterozygous mice, indicating the crucial role of α-Klotho as a humoral factor regulating EC homeostasis [44]. Moreover, in cultured ECs α-Klotho also regulates the homeostatic balance between ROS and antioxidant agents, and decreases hydrogen peroxide-induced apoptosis and cellular senescence [36]. In addition, α-Klotho has a protective effect against angiotensin II-induced ROS production and reduces the activation of superoxide anions [45, 46]. Thus, the downregulation of α-Klotho in the microvascular endothelium might substantially contribute to ROS generation, oxidative stress and tissue damage in the skin of patients with SSc.

In mouse models, α-Klotho also regulates the BM microenvironment, including macrophages, fibroblasts, ECs and extracellular matrix, with potential effects on hematopoiesis and EPC differentiation [47]. The differentiation and mobilization of EPCs from the BM is likely impaired in *kl-/kl-* mice, as shown by decreased numbers of EPC-like cells both in the BM compartment and in the peripheral blood [48]. Although conflicting data have been reported on the role of EPC dysfunction in SSc [49, 50], further evaluation of the possible role of sKl in EPCs from patients with SSc could be of interest.

sKl has been recently shown to maintain EC homeostasis in association with VEGF_165_-bound VEGFR-2 and TRPC-1 by forming a heterotrimeric complex on the surface of ECs, which regulates Ca^2+^ influx, maintaining plasma membrane permeability and cell integrity [16]. As previously demonstrated [13], SSc-MVECs strongly express the anti-angiogenic VEGF_165_b isoform that binds VEGFR-2 with the same affinity of VEGF_165_ but does not activate proangiogenic downstream signaling cascades. Moreover, we showed here that TRPC-1 is downregulated in SSc-MVECs. Thus, either the downregulation of TRPC-1 or the preferential binding of the upregulated anti-angiogenic VEGF_165_b isoform to VEGFR-2 may prevent the formation of the VEGF_165_/VEGFR-2/TRPC-1/sKl molecular complex on the plasma membrane of SSc-MVECs, contributing to cell dysfunction and impairment of angiogenesis. Interestingly, here we also provide evidence that the exogenous addition of sKl to SSc-MVECs might improve the formation of the aforementioned complex through the downregulation of VEGF_165_b and the parallel upregulation of TRPC-1 with a consequent boost of the angiogenic response.

## Conclusions

In SSc, microvascular injury, chronic ischemia and tissue hypoxia lead to EC apoptosis and dysfunction, with several regulatory molecular pathways being involved in the pathogenic mechanisms underlying endothelial damage [51–55]. In this complex scenario, our study provides evidence that the administration of exogenous sKl may effectively improve SSc-MVEC functions in vitro by acting as a powerful proangiogenic factor fostering the formation of capillary-like tubes similar to those formed by H-MVECs. Further studies will be necessary to investigate how this pleiotropic protein may regulate the microvascular milieu in SSc and to clarify whether sKl administration might be useful to counteract SSc-related endothelial dysfunction. On the basis of our in vitro data, it will be of primary importance to test the potential utility of sKl peptide analogs as a novel therapeutic option to slow down the microvascular injury in patients with SSc.

## Abbreviations

ADAM: A disintegrin and metalloprotease
BM: Bone marrow
BSA: Bovine serum albumin
dcSSc: Diffuse cutaneous systemic sclerosis
EBM: Endothelial cell basal medium
ECs: Endothelial cells
EPCs: Endothelial precursor cells
FBS: Fetal bovine serum
FGF23: Fibroblast growth factor 23
H-MVECs: Healthy microvascular endothelial cells
kDa: kiloDalton
lcSSc: Limited cutaneous systemic sclerosis
MVECs: Microvascular endothelial cells
NO: Nitric oxide
PBS: Phosphate-buffered saline
ROS: Reactive oxygen species
SD: Standard deviation
SEM: Standard error of the mean
sKl: Soluble α-Klotho
SSc: Systemic sclerosis
SSc-MVECs: Systemic sclerosis microvascular endothelial cells
tKl: Transmembrane α-Klotho
TRPC-1: Transient receptor potential canonical-1
VEGF: Vascular endothelial growth factor
VEGFR-2: Vascular endothelial growth factor receptor-2

## References

[1] Balbir-Gurman A, Braun-Moscovici Y (2012). Scleroderma - new aspects in pathogenesis and treatment. Best Pract Res Clin Rheumatol.

[2] LeRoy EC, Black C, Fleischmajer R, Jablonska S, Krieg T, Medsger TA (1988). Scleroderma (systemic sclerosis): classification, subsets and pathogenesis. J Rheumatol.

[3] van den Hoogen F, Khanna D, Fransen J, Johnson SR, Baron M, Tyndall A (2013). 2013 Classification criteria for systemic sclerosis: an American College of Rheumatology/European League Against Rheumatism Collaborative initiative. Arthritis Rheum.

[4] Manetti M, Guiducci S, Ibba-Manneschi L, Matucci-Cerinic M (2011). Impaired angiogenesis in systemic sclerosis: the emerging role of the antiangiogenic VEGF165b splice variant. Trends Cardiovasc Med.

[5] Carmeliet P (2005). Angiogenesis in life, disease and medicine. Nature.

[6] Folkman J (1995). Angiogenesis in cancer, vascular, rheumatoid and other disease. Nat Med.

[7] Rafii S (2000). Circulating endothelial precursors: mystery, reality, and promise. J Clin Invest.

[8] Isner JM, Asahara T (1999). Angiogenesis and vasculogenesis as therapeutic strategies for postnatal neovascularization. J Clin Invest.

[9] Asahara T, Murohara T, Sullivan A, Silver M, van der Zee R, Li T (1997). Isolation of putative progenitor endothelial cells for angiogenesis. Science.

[10] Cooper LT, Cooke JP, Dzau VJ (1994). The vasculopathy of aging. J Gerontol.

[11] De Santis M, Ceribelli A, Cavaciocchi F, Crotti C, Massarotti M, Belloli L (2016). Nailfold videocapillaroscopy and serum VEGF levels in scleroderma are associated with internal organ involvement. Auto Immun Highlights.

[12] Bielecki M, Kowal K, Lapinska A, Chwiesko-Minarowska S, Chyczewski L, Kowal-Bielecka O (2011). Peripheral blood mononuclear cells from patients with systemic sclerosis spontaneously secrete increased amounts of vascular endothelial growth factor (VEGF) already in the early stage of the disease. Adv Med Sci.

[13] Manetti M, Guiducci S, Romano E, Ceccarelli C, Bellando-Randone S, Conforti ML (2011). Overexpression of VEGF165b, an inhibitory splice variant of vascular endothelial growth factor, leads to insufficient angiogenesis in patients with systemic sclerosis. Circ Res.

[14] Choi JJ, Min DJ, Cho ML, Min SY, Kim SJ, Lee SS (2003). Elevated vascular endothelial growth factor in systemic sclerosis. J Rheumatol.

[15] Distler O, Distler JH, Scheid A, Acker T, Hirth A, Rethage J (2004). Uncontrolled expression of vascular endothelial growth factor and its receptors leads to insufficient skin angiogenesis in patients with systemic sclerosis. Circ Res.

[16] Kusaba T, Okigaki M, Matui A, Murakami M, Ishikawa K, Kimura T (2010). Klotho is associated with VEGF receptor-2 and the transient receptor potential canonical-1 Ca2+ channel to maintain endothelial integrity. Proc Natl Acad Sci U S A.

[17] Matsumura Y, Aizawa H, Shiraki-Iida T, Nagai R, Kuro-o M, Nabeshima Y (1998). Identification of the human Klotho gene and its two transcripts encoding membrane and secreted Klotho protein. Biochem Biophys Res Commun.

[18] Shiraki-Iida T, Aizawa H, Matsumura Y, Sekine S, Iida A, Anazawa H (1998). Structure of the mouse klotho gene and its two transcripts encoding membrane and secreted protein. FEBS Lett.

[19] Kuro-o M, Matsumura Y, Aizawa H, Kawaguchi H, Suga T, Utsugi T (1997). Mutation of the mouse Klotho gene leads to a syndrome resembling ageing. Nature.

[20] Kurosu H, Yamamoto M, Clark JD, Pastor JV, Nandi A, Gurnani P (2005). Suppression of aging in mice by the hormone Klotho. Science.

[21] Imura A, Iwano A, Tohyama O, Tsuji Y, Nozaki K, Hashimoto N (2004). Secreted Klotho protein in sera and CSF: implication for post-translational cleavage in release of Klotho protein from cell membrane. FEBS Lett.

[22] Li SA, Watanabe M, Yamada H, Nagai A, Kinuta M, Takei K (2004). Immunohistochemical localization of Klotho protein in brain, kidney, and reproductive organs of mice. Cell Struct Funct.

[23] Kurosu H, Ogawa Y, Miyoshi M, Yamamoto M, Nandi A, Rosenblatt KP (2006). Regulation of fibroblast growth factor-23 signaling by klotho. J Biol Chem.

[24] Kuro-o M (2006). Klotho as a regulator of fibroblast growth factor signaling and phosphate/calcium metabolism. Curr Opin Nephrol Hypertens.

[25] Hu MC, Kuro-o M, Moe OW (2012). The emerging role of Klotho in clinical nephrology. Nephrol Dial Transplant.

[26] Yamamoto M, Clark JD, Pastor JV, Gurnani P, Nandi A, Kurosu H (2005). Regulation of oxidative stress by the anti-aging hormone klotho. J Biol Chem.

[27] Hsieh CC, Kuro-o M, Rosenblatt KP, Brobey R, Papaconstantinou J (2010). The ASK1-Signalosome regulates p38 MAPK activity in response to levels of endogenous oxidative stress in the Klotho mouse models of aging. Aging (Albany NY).

[28] Kuro-o M (2012). Klotho in health and disease. Curr Opin Nephrol Hypertens.

[29] Chen CD, Podvin S, Gillespie E, Leeman SE, Abraham CR (2007). Insulin stimulates the cleavage and release of the extracellular domain of Klotho by ADAM10 and ADAM17. Proc Natl Acad Sci U S A.

[30] Ohyama Y, Kurabayashi M, Masuda H, Nakamura T, Aihara Y, Kaname T (1998). Molecular cloning of rat klotho cDNA: markedly decreased expression of klotho by acute inflammatory stress. Biochem Biophys Res Commun.

[31] Vervloet MG, Larsson TE (2011). Fibroblast growth factor-23 and Klotho in chronic kidney disease. Kidney Int.

[32] Wang Y, Kuro-o M, Sun Z (2012). Klotho gene delivery suppresses Nox2 expression and attenuates oxidative stress in rat aortic smooth muscle cells via the cAMP-PKA pathway. Aging Cell.

[33] Scott PA, Bicknell R (1993). The isolation and culture of microvascular endothelium. J Cell Sci.

[34] D’Alessio S, Fibbi G, Cinelli M, Guiducci S, Del Rosso A, Margheri F (2004). Matrix metalloproteinase 12-dependent cleavage of urokinase receptor in systemic sclerosis microvascular endothelial cells results in impaired angiogenesis. Arthritis Rheum.

[35] Margheri F, Manetti M, Serratì S, Nosi D, Pucci M, Matucci-Cerinic M (2006). Domain 1 of the urokinase-type plasminogen activator receptor is required for its morphologic and functional, beta2 integrin-mediated connection with actin cytoskeleton in human microvascular endothelial cells: failure of association in systemic sclerosis endothelial cells. Arthritis Rheum.

[36] Ikushima M, Rakugi H, Ishikawa K, Maekawa Y, Yamamoto K, Ohta J (2006). Anti-apoptotic and anti-senescence effects of klotho on vascular endothelial cells. Biochem Res Commun.

[37] Maekawa Y, Ohishi M, Ikushima M, Yamamoto K, Yasuda O, Oguro R (2011). Klotho protein diminishes endothelial apoptosis and senescence via a mitogen-activated kinase pathway. Geriatric Gerontol Int.

[38] de Oliveira RM (2006). Klotho RNAi induces premature senescence of human cells via a p53/p21 dependent pathway. FEBS Lett.

[39] Safirstein R, DiMari J, Megyesi J, Price P. Mechanisms of renal repair and survival following acute injury. Semin Nephrol. 1998;18:519–22. 9754604

[40] Rubin H (1997). Cell aging in vivo and in vitro. Mech Ageing Dev.

[41] Imura A, Tsuji Y, Murata M, Maeda R, Kubota K, Iwano A (2007). Alpha-Klotho as a regulator of calcium homeostasis. Science.

[42] Griffith TM, Edwards DH, Lewis MJ, Newby AC, Henderson AH (1984). The nature of endothelium-derived vascular relaxant factor. Nature.

[43] Palmer RM, Ferrige AG, Moncada S (1987). Nitric oxide release accounts for the biological activity of endothelium-derived relaxing factor. Nature.

[44] Saito Y, Nakamura T, Ohyama Y, Suzuki T, Iida A, Shiraki-Iida T (2000). In vivo klotho gene delivery protects against endothelial dysfunction in multiple risk factor syndrome. Biochem Biophys Res Commun.

[45] Rakugi H, Matsukawa N, Ishikawa K, Yang J, Imai M, Ikushima M (2007). Anti-oxidative effect of Klotho on endothelial cells through cAMP activation. Endocrine.

[46] Mukai Y, Shimokawa H, Higashi M, Morikawa K, Matoba T, Hiroki J (2002). Inhibition of renin-angiotensin system ameliorates endothelial dysfunction associated with aging in rats. Arterioscler Thromb Vasc Biol.

[47] Okada S, Yoshida T, Hong Z, Ishii G, Hatano M (2000). Kuro-OM, et al. Impairment of B lymphopoiesis in precocious aging (klotho) mice. Int Immunol.

[48] Shimada T, Takeshita Y, Murohara T, Sasaki K, Egami K, Shintani S (2004). Angiogenesis and vasculogenesis are impaired in the precocious-aging klotho mouse. Circulation.

[49] Kuwana M, Okazaki Y, Yasuoka H, Kawakami Y, Ikeda Y (2004). Defective vasculogenesis in systemic sclerosis. Lancet.

[50] Avouac J, Juin F, Wipff J, Couraud PO, Chiocchia G, Kahan A (2008). Circulating endothelial progenitor cells in systemic sclerosis: association with disease severity. Ann Rheum Dis.

[51] Manetti M, Guiducci S, Ibba-Manneschi L, Matucci-Cerinic M (2010). Mechanisms in the loss of capillaries in systemic sclerosis: angiogenesis versus vasculogenesis. J Cell Mol Med.

[52] Cipriani P, Guiducci S, Miniati I, Cinelli M, Urbani S, Marrelli A (2007). Impairment of endothelial cell differentiation from bone marrow-derived mesenchymal stem cells: new insight into the pathogenesis of systemic sclerosis. Arthritis Rheum.

[53] Mold C, Morris CA (2001). Complement activation by apoptotic endothelial cells following hypoxia/reoxygenation. Immunology.

[54] Kahaleh MB (2004). Vascular involvement in systemic sclerosis. Clin Exp Rheumatol.

[55] Manetti M, Guiducci S, Matucci-Cerinic M (2016). The crowded crossroad to angiogenesis in systemic sclerosis: where is the key to the problem?. Arthritis Res Ther.

